# *Meplitumenaluna* gen. nov., sp. nov. an interesting eutardigrade (Hypsibiidae, Itaquasconinae) from the Sierra Nevada de Santa Marta, Colombia

**DOI:** 10.3897/zookeys.865.30705

**Published:** 2019-07-22

**Authors:** Oscar Lisi, Anisbeth Daza, Rosana Londoño, Sigmer Quiroga, Giovanni Pilato

**Affiliations:** 1 Dipartimento di Scienze Biologiche, Geologiche e Ambientali, Università di Catania, Via Androne 81, 95124 Catania, Italy; 2 Grupo de Investigación MIKU, Universidad del Magdalena, Carrera 32 # 22-08, Santa Marta DTCH, Colombia; 3 Facultad de Ciencias Básicas, Programa de Biología, Universidad del Magdalena, Carrera 32 # 22-08, Santa Marta DTCH, Colombia

**Keywords:** Magdalena, phylogeny, Tardigrada, taxonomy, water bear

## Abstract

A new genus of Itaquasconinae, *Meplitumen***gen. nov.**, and a new species, *Meplitumenaluna***sp. nov.**, are described. The new genus has characters present in other genera of Itaquasconinae but in a unique combination. The spiral thickening of the bucco-pharyngeal tube is also present anteriorly to the insertion point of the stylet supports, excluding only the short portion where the apophyses for the insertion of the stylet muscles (AISM) are present. This character is similar to *Astatumen* Pilato, 1997 but *Meplitumen***gen. nov.** differs from this genus as stylet furcae are shaped differently and as stylet supports and placoids are present. The presence of a spiral thickening in a portion of the buccal tube anterior to the stylet supports distinguishes the new genus from *Mesocrista* Pilato, 1987, *Platicrista* Pilato, 1987 and *Itaquascon* de Barros, 1939. *Meplitumen***gen. nov.** also differs from *Mesocrista*, in having the caudal processes of the AISM pointing laterally (instead of postero-laterally), and the apices of the caudal processes of the stylet furcae unswollen. From *Itaquascon* the new genus also differs by having more robust stylet supports, pharyngeal bulb with placoids, stylet furcae differently shaped. *Meplitumen***gen. nov.** also differs from *Platicrista* in having caudal processes of the AISM more robust and not flexible, and more slender stylet supports. The new species, *Meplitumenaluna***sp. nov.**, has a cuticle with a very faint roughness at the caudal extremity of the body, and eyes probably absent. The pharyngeal bulb is long, with two long, narrow, rod-shaped macroplacoids; a microplacoid and septulum are absent. The claws are well developed with main branches provided with accessory points, and at the base of the claws, a structure interpretable as a very thin lunule is present. Other cuticular thickenings on the legs are absent.

## Introduction

Rarely in the study of systematics and phylogeny is one so fortunate to encounter a taxon with characters that sheds light on phylogenetic directions within a group. While studying Colombian tardigrades, from the Sierra Nevada de Santa Marta, we found eight specimens and two exuviae with eggs of a new species of Hypsibiidae (Itaquasconinae), for which it is necessary to erect a new genus that we name *Meplitumen* gen. nov. This discovery is further confirmation of the biodiversity richness of this Neotropical region (Myers et al. 2000, Rull 2007), which, at least with regards to the tardigrades, is still far from being thoroughly investigated.

In the framework of the subfamily Itaquasconine Rudescu, 1964, the four genera *Itaquascon* de Barros, 1939, *Mesocrista* Pilato, 1987, *Platicrista*, Pilato, 1987 and *Astatumen* Pilato, 1997 are certainly related to one another, but until now it has not been possible to formulate any hypothesis about the phylogenetic relationships that exist among them. Our new genus shows characters that are present in these other genera but in a unique combination. This encouraged us to hypothesise possible evolutionary pathways that connect these five Itaquasconinae genera, which appear to constitute a homogeneous group within the subfamily.

## Material and methods

The present work is part of a revision of the tardigradological collection of the Centro de Colecciones Biológicas de la Universidad del Magdalena (CBUMAG:TAR), Santa Marta, Colombia. The material studied with the new genus and species has been returned to the collection.

The original specimens had been extracted from moss and lichen samples collected in San Lorenzo and El Campano, Colombia. Complete information about localities and samples studied are included in the description of the new species under Material examined.

The studied specimens were mounted in polyvinyl alcohol mounting media (BioQuip #6371A). Measurements are given in micrometres (µm), and photomicrographs made under ×100 oil immersion under phase contrast and differential interference contrast microscopy, using a Leica “DMLB” Microscope equipped with “Canon S40” digital camera and a micrometre, a Zeiss Axio Scope A1 with CCD camera Zeiss AxioCam ICc5, and a Zeiss AxioLab A1 with a Zeiss Axiocam ERc 5s. Images were edited, and plates arranged, using Adobe Photoshop Elements 2.0 digital imaging software.

**Figure 1.**
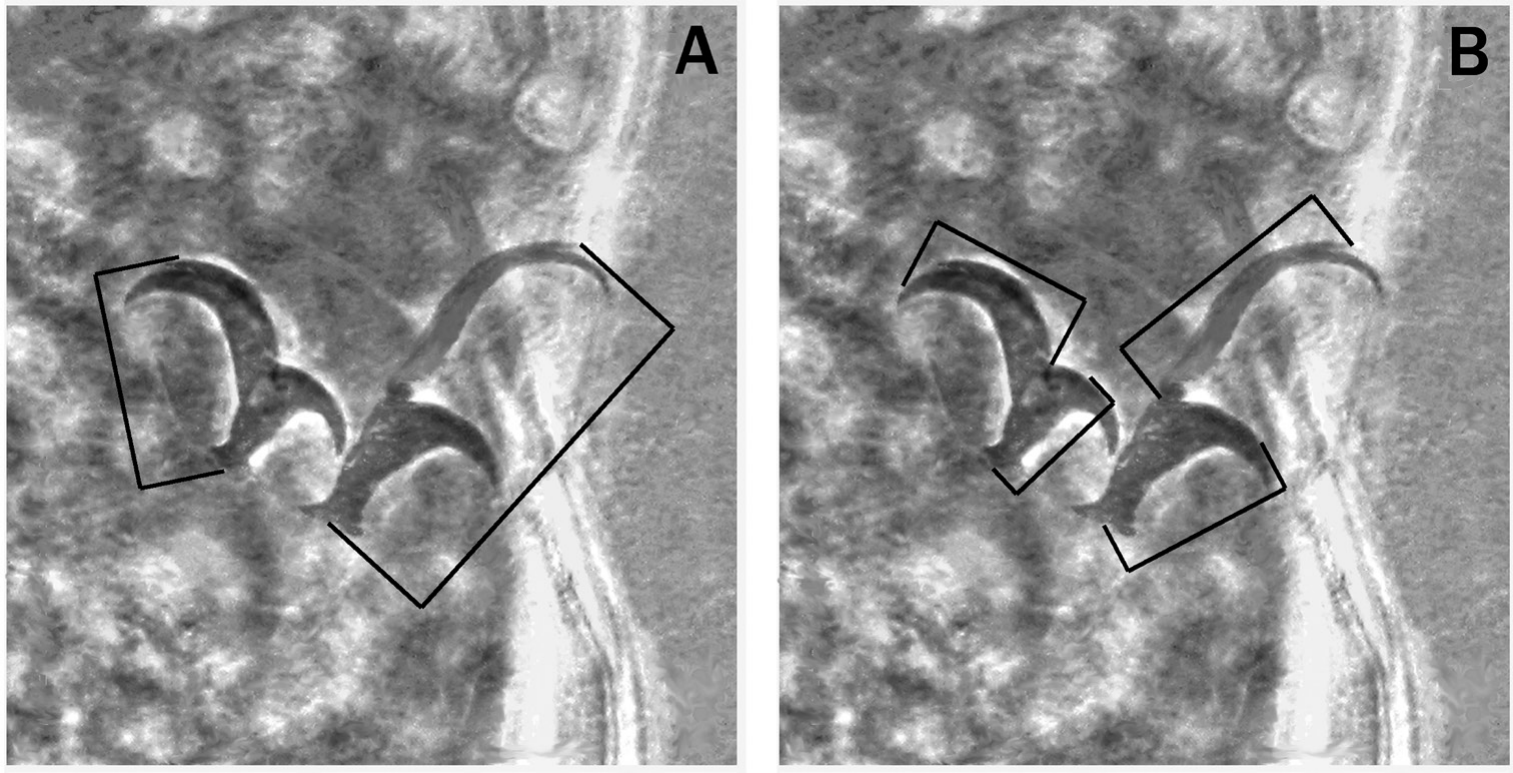
Claws of the *Hypsibius* type showing the criteria used to take the measurements in *Meplitumenaluna* gen. nov., sp. nov. **A** claw orientation chosen for measuring the entire length **B** claw orientation and referring points chosen for various claw portion measurement. The claws shown, used as example, belong to *Platicristaangustata* (Murray, 1905).

Notwithstanding the presence of spiral thickening, we refer to the “buccal tube” as the entire portion of the bucco-pharyngeal tube anterior to the stylet supports, which seems to be almost rigid. A problem of interpretation and terminology with regard to that portion of the bucco-pharyngeal tube is discussed in the Appendix 1 of this paper. The *pbf* index is the percent ratio between the length of the buccal tube and the total length of the bucco-pharyngeal tube (Pilato et al. 2002). The *pt* index is the percent ratio between the length of a structure and the length of the buccal tube measured from the anterior margin of the stylet sheaths to its posterior end (Pilato 1981): in the new genus, as in others, the posterior end of the buccal tube fixed to assess the *pt* index coincides with the insertion point of the stylet supports. Claws were measured using maximum length of the studied structures and choosing only those with the same, or at least very similar, orientation; we discarded claws with unsuitable orientation for correct measurement. As a consequence, the number of claws measured, reported in Table 1, is limited. When the claw measurement included the basal portion, we chose (Fig. 1A, B) the central point of the claw base and the most distant point of the primary or secondary claw branch, including accessory points. In measuring the main branch of the external claws (Fig. 1B), we were unable to measure only the sclerified portion excluding the very flexible basal section as the precise border was not always clearly defined. That flexible portion may be more or less bent and then its exclusion would be preferable in order to obtain more precise measurements, but it was not possible for us to avoid this problem. The main branch of the internal claws was measured from the junction point of primary and secondary branch (Fig. 1B) to the more distant point of the primary branch.

**Table 1.** Morphological measurements for the holotype, five paratypes, and the additional specimen of *Meplitumenaluna* sp. nov. The first three small specimens are probably juveniles or males. Measurements given in µm, with **pbf** and **pt** index values for relevant structures, and the percent ratio between the main branch and the total length of the external claw. Specimens ordered by buccal tube length (body length being a less precise measurement). The small specimens differ from the larger in the **pt** values relative to the buccal tube width, the macroplacoid length, and claw II and III length (less remarkably claw IV length), but the percent values of the main branch length with respect to the total claw lengths are compatible.

No. slide	00376	00477	00476	00461	00545	00462 holotype	00460
Body length	303	258	265	482	486	590	515
Bucco-pharyngeal tube length	40.6	41.6	42.4	60.0	61.2	61.3	?
Buccal tube length	21.0	23.3	23.6	30.6	31.7	31.8	33.7
pbf	51.7	56.0	55.7	51.0	51.8	51.9	?
Buccal tube external width	5.2	5.6	5.7	8.7	8.8	8.9	9.5
pt	24.8	24.0	24.2	28.4	27.8	28.0	28.2
pt stylet supports insertion point	100	100	100	100	100	100	100
First placoid	?	5.2	5.2	9.3	10.7	10.9	10.6
pt	?	22.3	22.0	30.4	33.7	34.3	31.4
Second placoid	?	14.3	14.5	25.6	29.0	29.0	29.0
pt	?	61.4	61.4	83.7	91.5	91.2	86.1
Placoid row	?	20.3	20.4	34.5	40.2	40.7	40.7
pt	?	87.1	86.4	112.7	126.8	128.0	120.8
External claw I	14.5	?	15.4	21.4	21.3	?	?
pt	69.0	?	65.3	69.9	67.2	?	?
External claw I - main branch	10.4	?	11.3	15.0	15.2	?	?
pt	49.5	?	47.9	49.0	47.9	?	?
External claw I - main branch % total	71.7	?	73.4	70.1	71.4	?	?
External claw I - base + secondary branch	6.7	?	7.3	11.3	11.8	12.1	?
pt	32.2	?	30.9	36.9	37.2	38.1	?
Internal claw I	?	?	9.7	14.0	13.7	?	?
pt	?	?	41.1	45.7	43.2	?	?
Internal claw I - main branch	?	?	6.1	8.6	8.5	?	?
pt	?	?	25,8	28.1	26.8	?	?
Internal claw I - main branch % total	?	?	62.9	61.4	62.0	?	?
Internal claw I - base + secondary branch	?	6.2	6.1	9.0	9.1	10.2	?
pt	?	26.6	25.8	29.4	28.7	32.1	?
External claw II	15.9	16.9	?	?	?	29.0	?
pt	76.4	72.5	?	?	?	91.2	?
External claw II - main branch	11.5	12.2	?	?	?	20.0	?
pt	55.3	52.4	?	?	?	62.9	?
External claw II - main branch % total	72.3	72.2	?	?	?	69.0	?
External claw II - base + secondary branch	7.1	?	7.4	?	?	13.8	?
pt	34.1	?	31.3	?	?	43.4	?
Internal claw II	?	10.5	10.9	?	?	18.4	?
pt	?	45.1	46.2	?	?	57.9	?
Internal claw II - main branch	?	7.5	7.7	?	?	12.9	?
pt	?	32.2	32.6	?	?	40.2	?
Internal claw II - main branch % total	?	71.4	70.6	?	?	70.1	?
Internal claw II - base + secondary branch	?	6.6	6.3	?	?	11.2	?
pt	?	28.3	26.7	?	?	35.2	?
External claw III	?	18.1	17.4	?	?	28.6	29.0
pt	?	77.7	73.7	?	?	89.9	86.1
External claw III - main branch	?	13.0	11.8	?	18.2	19.5	19.6
pt	?	55.8	50.0	?	57.4	61.3	58.2
External claw III - main branch % total	?	71.8	67.8	?	?	68.2	67.6
pt	?	36.1	33.1	38.6	?	42.1	39.2
Internal claw III	10.3	10.9	?	?	17.4	19.3	?
pt	49.5	46.8	?	?	54.9	60.7	?
Internal claw III - main branch	?	7.3	?	?	11.4	12.7	?
pt	?	31.3	?	?	36.0	39.9	?
Internal claw III - main branch % total	?	67.0	?	?	65.5	65.8	?
Internal claw III - base + secondary branch	?	6.6	6.3	?	?	11.4	11.1
pt	?	28.3	26.7	?	?	35.8	32.9
Posterior claw IV	?	20.0	20.2	?	?	30.5	30.5
pt	?	85.8	85.6	?	?	95.9	90.5
Posterior claw IV - main branch	?	14.5	14.1	?	?	21.8	21.3
pt	?	62.2	59.7	?	?	68.6	63.2
Posterior claw IV - main branch % total	?	69.0	70.1	?	?	71.5	69.8
Posterior claw IV - base + secondary branch	?	9.8	10.1	14.0	?	15.3	14.7
pt	?	42.1	42.8	45.8	?	48.1	43.6
Anterior claw IV	?	10.9	11.0	16.2	?	17.6	18.1
pt	?	46.8	46.6	52.9	?	55.3	53.7
Anterior claw IV - main branch	?	7.7	7.7	11.3	?	12.1	12.2
pt	?	33.0	32.6	36.9	?	38.0	36.2
Anterior claw IV - main branch % total	?	70.6	70.0	69.7	?	68.7	67.4
Anterior claw IV - base + secondary branch	?	?	9.1	12.7	?	13.6	13.0
pt	?	?	38.6	41.5	?	42.8	38.6

For comparison, we have examined specimens of: *Mesocristaspitzbergensis* (Richters, 1903) from Tatra Mountains (border between Poland and Slovakia); *Platicristaangustata* (Murray, 1905) from Ligorzano (Modena, Italy); *Itaquasconcambewarrense* Pilato, Binda & Claxton, 2002 from Cambewarra Mountain (Australia), and types of *Astatumentrinacriae* (Arcidiacono, 1962) from Nebrodi Mounts (Sicily). All these specimens are deposited in the Binda and Pilato collection (Museum of the Department of Biological, Geological and Environmental Sciences, University of Catania, Italy).

For phylogenetic analysis, a character matrix was prepared and a parsimony analysis applied with a Nearest Neighbor Interchange (NNI) heuristic method, using the software Mesquite (Maddison and Maddison).

## Results

### Taxonomic account

#### Phylum Tardigrada Doyère, 1840

##### Class Eutardigrada Richters, 1926

###### Order Parachela Schuster, Nelson, Grigarick & Christenberry, 1980

####### Superfamily Hypsibioidea Pilato, 1969^1^

######## Family Hypsibiidae Pilato, 1969^2^

######### Subfamily Itaquasconinae Rudescu, 1964^3^

########## Meplitumen gen. nov.

Taxon classificationAnimaliaParachelaHypsibiidae

3c39ea68-5904-5296-a8df-8e8b205635f1

http://zoobank.org/4842673D-73A5-459A-A9DC-11C667772F10

**Figure 2.**
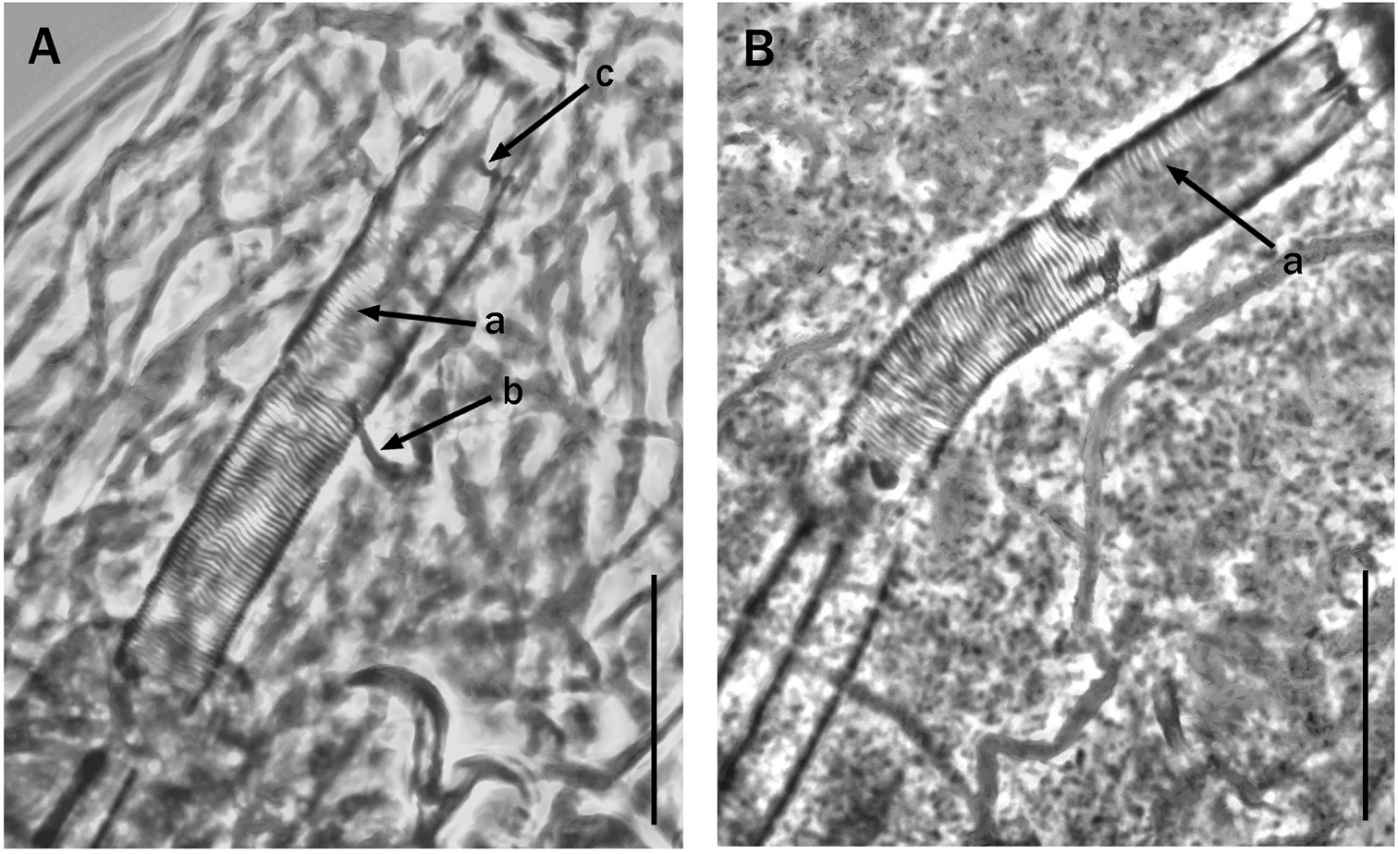
Bucco-pharyngeal tube of *Meplitumenaluna* gen. nov., sp. nov. **A** holotype (slide No. 00462), was invaded by fungal hyphae. The spiral thickening also anterior to the stylet supports insertion point is visible (arrow ‘a’). The arrow ‘b’ indicates the stylet supports. Arrow ‘c’ indicates the caudal processes pointing sideways of the wide and flat AISM**B** paratype (slide No. 00460). The spiral thickening also anterior to the stylet supports insertion point is visible (arrow ‘a’). Scale bar: 20 µm.

**Figure 3.**
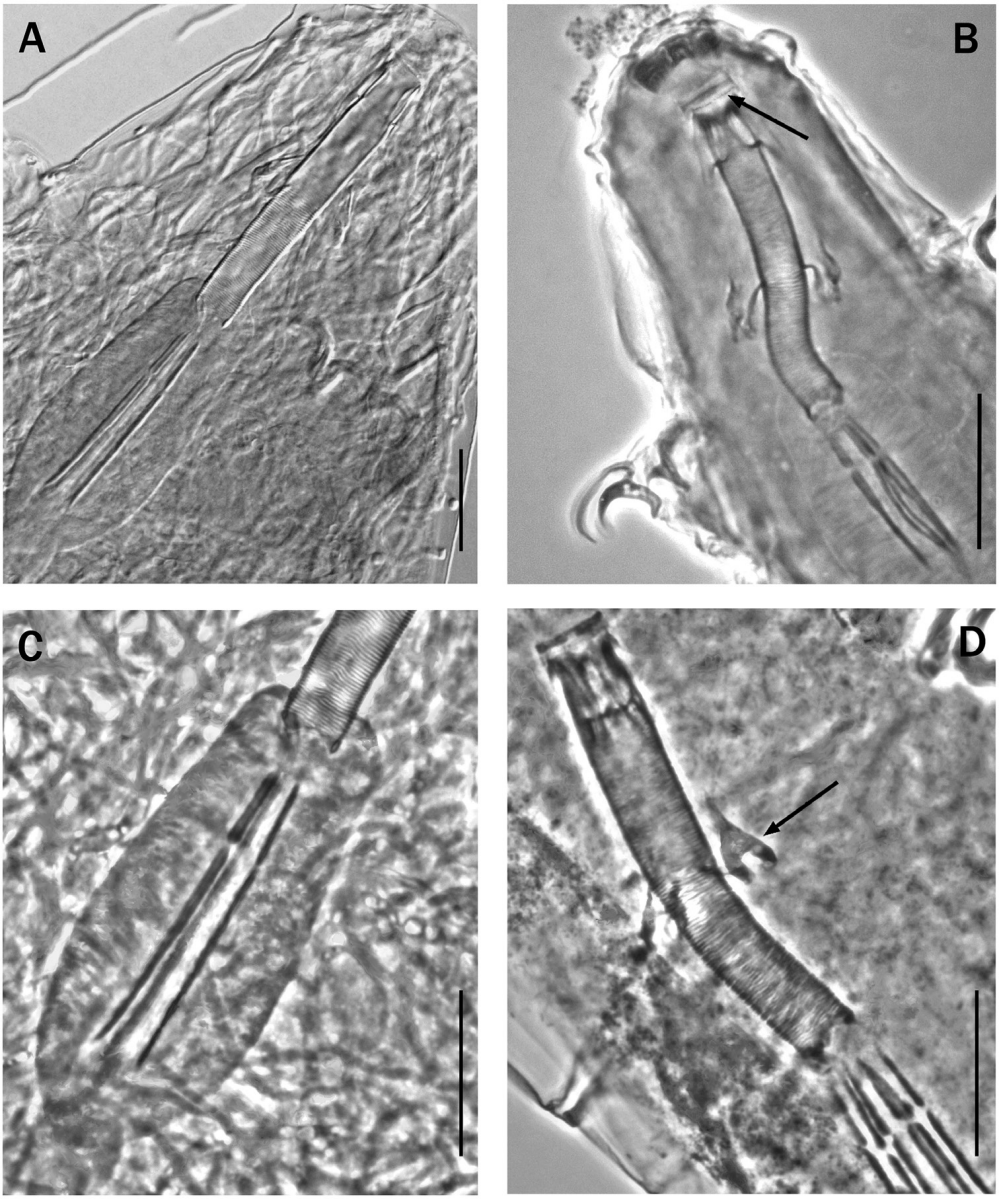
Bucco-pharyngeal apparatus of *Meplitumenaluna* gen. nov., sp. nov. **A** holotype **B** paratype (slide No. 00477); the arrow indicates a row of small teeth **C** detail of the pharyngeal bulb of the holotype with the long placoids well visible **D** bucco-pharyngeal apparatus of a paratype (slide No. 00461); the arrow indicates a stylet furca. Scale bar: 20 µm.

Figs 2 , 3 , 5 , 6A–C , 7

########### Type species.

*Meplitumenaluna* sp. nov.

########### Description.

Claws of the *Hypsibius*-type. Bucco-pharyngeal apparatus of Itaquasconinae model but buccal tube with a spiral thickening also present anteriorly to the stylet support insertion point (Fig. 2 A, B arrow ‘a’). Only the very anterior portion of the buccal tube, where the apophyses for the insertion of the stylet muscles (AISM) are present, is the spiral thickening absent. The AISM are wide and flat ridges, symmetrical with respect to the frontal plane (Figs 2A; 3A, B, D) and their caudal processes point sideways. Peribuccal lamellae absent, papulae probably present (needs confirmation). No cuticular thickening is present between buccal and pharyngeal tube (Figs 2A, B; 3A, B). The stylet furcae have the caudal processes with non-swollen apices (Fig. 3D, arrow). The stylet supports are normally developed (Fig. 2A, arrow ‘b’). The pharyngeal bulb is long, without apophyses but with true, long placoids (Fig. 3A–C).

########### Differential diagnosis.

**Figure 4.**
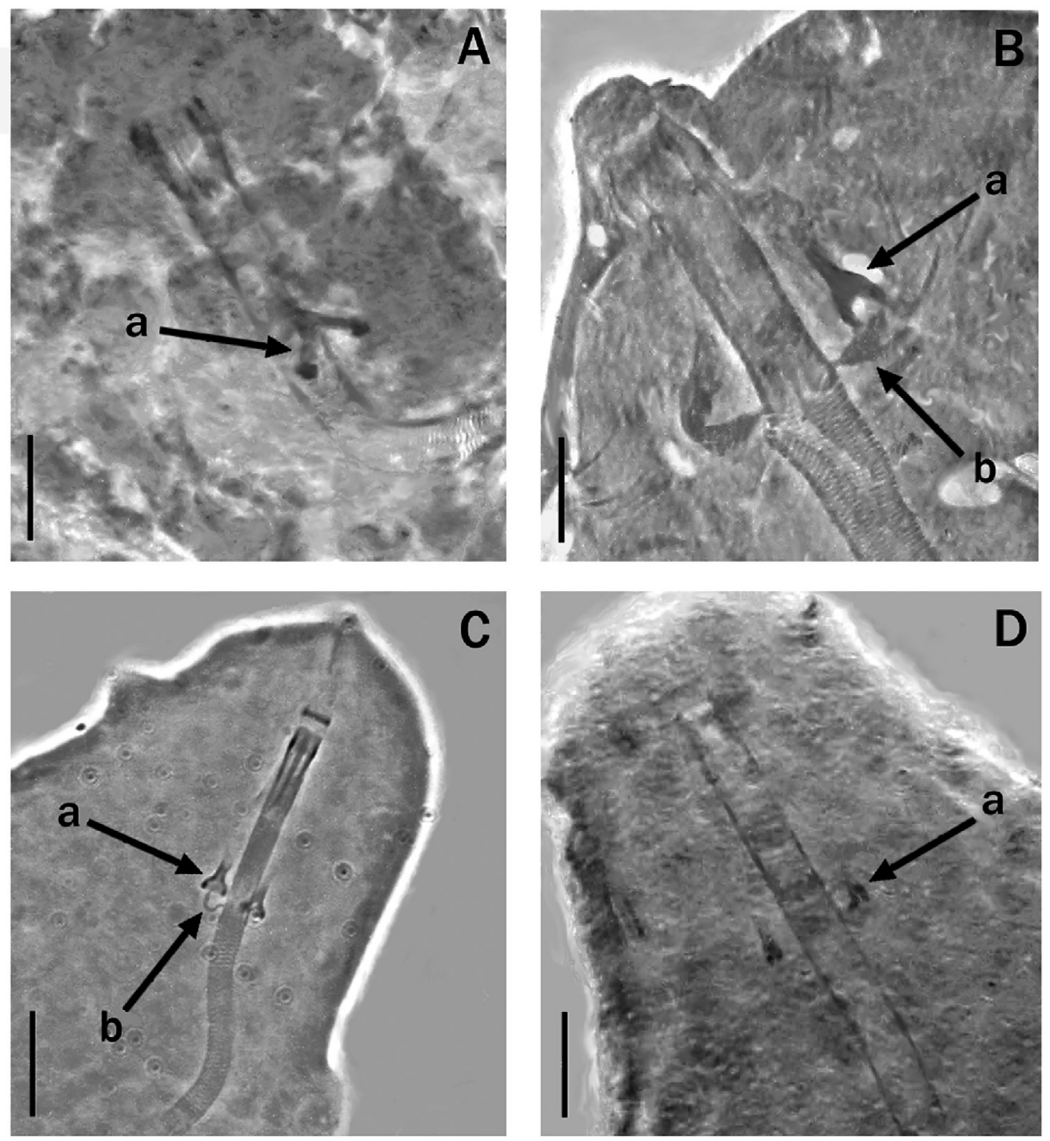
Shape of the stylet furca (arrow ‘a’) in species of various genera of Itaquasconinae**A***Mesocristaspitzbergensis* (Richters, 1903) **B***Platicristaangustata* (Murray, 1905) **C***Itaquasconcambewarrense* Pilato, Binda & Claxton, 2002 **D***Astatumentrinacriae* (Arcidiacono, 1962). Arrow ‘b’ indicates the stylet supports. Scale bar: 10 µm.

According to the definition in Pilato and Binda (2010), the bucco-pharyngeal tube of Itaquasconinae model, is subdivided into an anterior portion (buccal tube), rigid, without spiral thickening and without ventral lamina, and a posterior portion, generally flexible, provided with a spiral thickening (pharyngeal tube), as in *Astatumen*, *Mesocrista*, *Platicrista* and *Itaquascon*. In *Meplitumen* gen. nov. the bucco-pharyngeal apparatus differs from the above by having a spiral thickening also present anteriorly to the stylet support insertion point (Fig. 2A, B arrow ‘a’), which is similar to *Astatumen* but, unlike *Astatumen*, the stylet supports are present (Fig. 2A, arrow ‘b’), and mark the border between the posterior flexible portion of bucco-pharyngeal tube and the (almost) rigid anterior section. The sideways pointing caudal processes of the AISM, are similar to *Platicrista*, *Itaquascon* and *Astatumen*, but differ from *Mesocrista* (in which they point postero-laterally). The non-swollen apices (Fig. 3D, arrow) of the stylet furcae caudal processes are more similar to *Platicrista* (Fig. 4B, arrow ‘a’) than *Mesocrista* where the apices are clearly swollen (Fig. 4A, arrow ‘a’). The stylet furcae are clearly larger than those of *Itaquascon* (Fig. 4C, arrow ‘a’) and *Astatumen* (Fig. 4D, arrow ‘a’) whose caudal processes are very reduced.

**Etymology**. Considering that the bucco-pharyngeal apparatus of the new genus has characters present in *Mesocrista*, *Platicrista*, *Itaquascon* and *Astatumen*, we have chosen *Meplitumen* as generic name using the first letters of the name of the first three genera (Me-, Pl-, It-) and the final part of the name *Astatumen* (-umen).

########## Meplitumen aluna sp. nov.

Taxon classificationAnimaliaParachelaHypsibiidae

3c39ea68-5904-5296-a8df-8e8b205635f1

http://zoobank.org/C96630F3-7A49-479E-8B14-5C00788CC0AD

**Figure 5.**
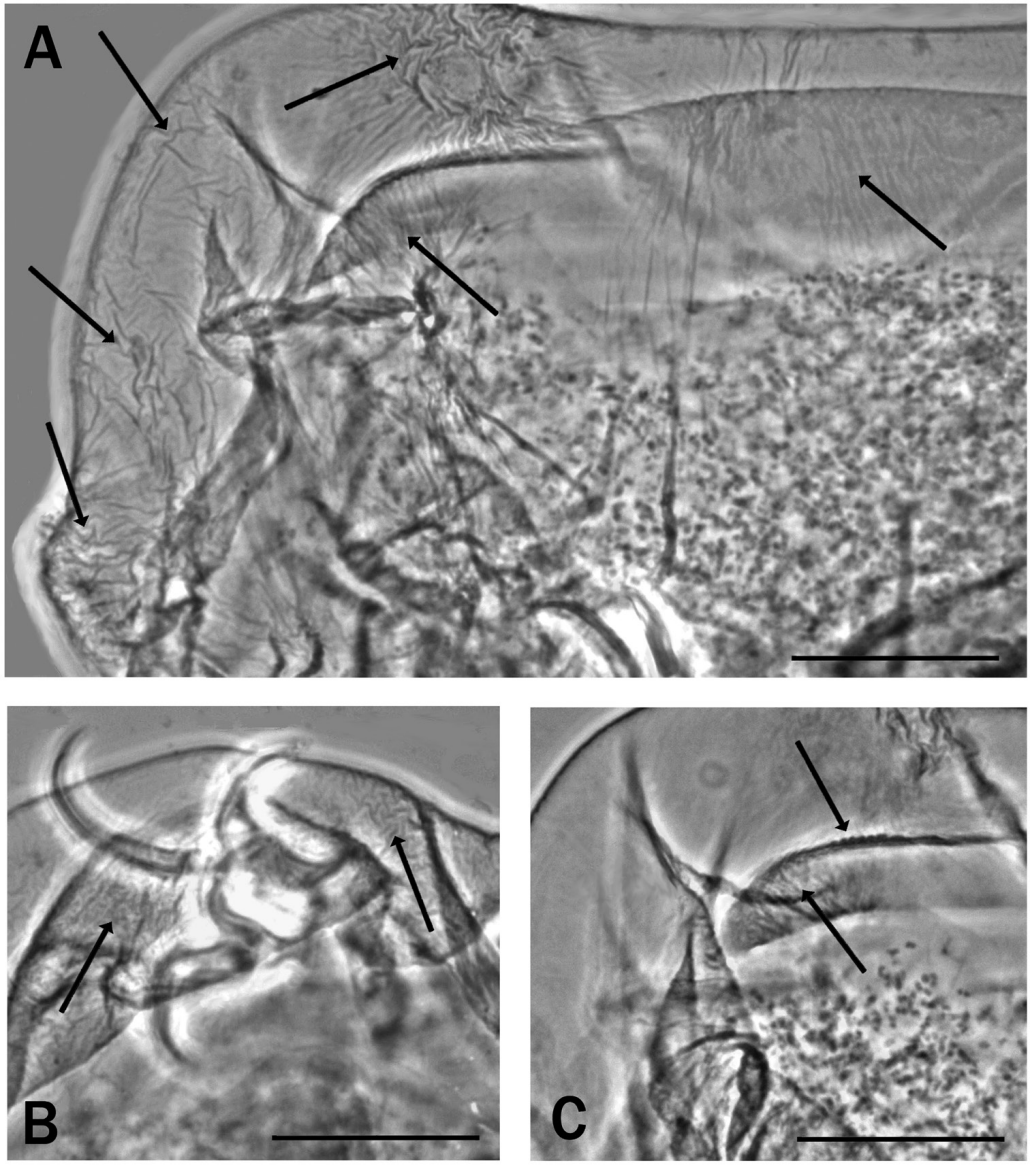
*Meplitumenaluna* gen. nov., sp. nov. Caudal extremity of the body where a faint roughness may be visible (arrows) **A** paratype (slide No. 00477) **B** and **C** paratype (slide No. 00460). Scale bar: 20 µm.

**Figure 6.**
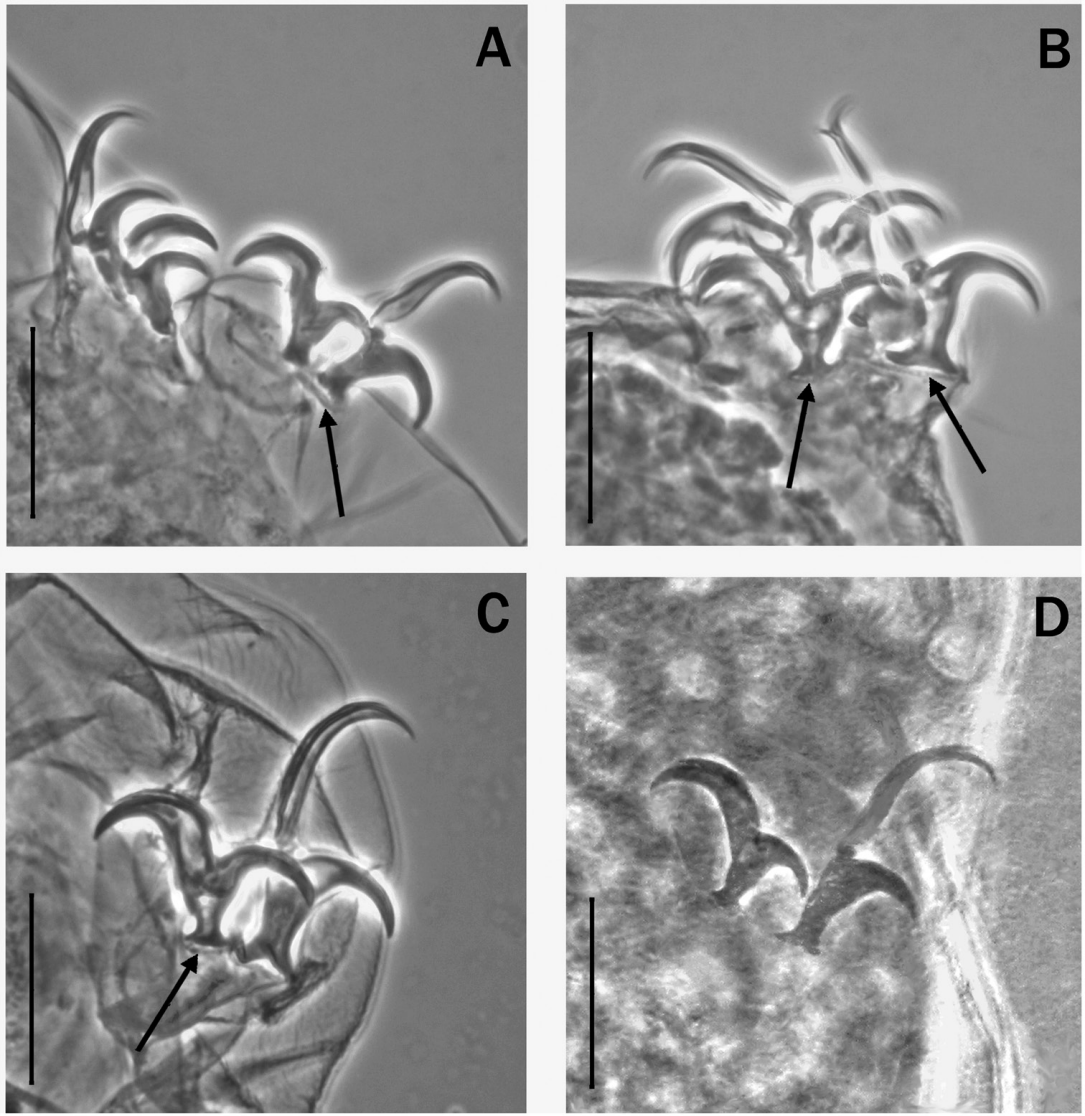
**A–C** claws of *Meplitumenaluna* sp. nov. (paratypes) **A** claws III **B** and **C** claws IV. The arrow indicates the structure interpretable as a very thin lunule **D** claws III of *Platicristaangustata* (Murray, 1905). It is evident that in *Meplitumenaluna* sp. nov. the claw secondary branches are longer and thinner than those of *Platicristaangustata*. Scale bar: 20 µm.

**Figure 7.**
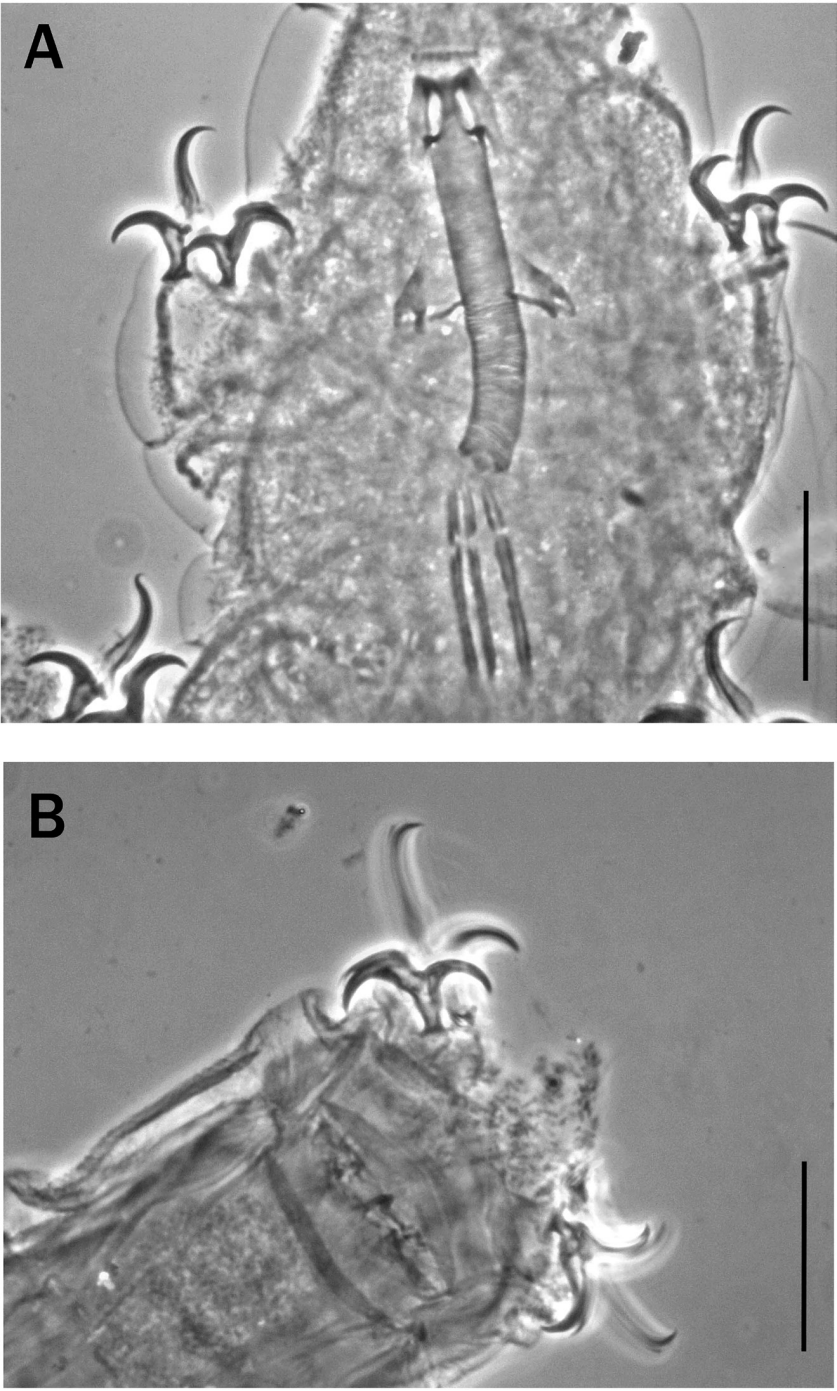
Small specimens (juvenile or male) of *Meplitumenaluna* sp. nov. **A** bucco-pharyngeal apparatus of a paratype (slide No. 00476) **B** claws of the hind legs of another paratype (slide No. 00477). Scale bar: 20 µm.

Figs 2 , 3 , 5 , 6A–C , 7 ; Table 1

########### Type locality.

San Lorenzo, Sierra Nevada de Santa Marta, Magdalena, Colombia.

########### Material examined.

**Holotype** (slide No. 00462), 5 **paratypes** (slides Nos. 00460, 00461, 00476, 00477), and two exuviae (slide No. 00492) from a sample dominated by lichens (*Usnea*, and *Parmotrema*) mixed with bryophytes (*Sematophyllum*, *Frullania*, *Microlejeunea*, and *Leucolejeunea*), collected in San Lorenzo (Colombia) at 11°06'16.9"N and 74°03'31.2"W, 2517 m a.s.l.. One paratype (slide No. 00545) collected in the type locality but in a different sample containing lichens (*Parmotrema, Heterodermia*, and *Hypotrachyna*) mixed with bryophytes (*Meteoridium*, *Frullania*, and *Archilejeunea*). One additional specimen (slide No. 00376) of a sample of liverworts (*Frullania* and *Cheilolejeunea*), collected in El Campano (Colombia) at 11°6'23.2"N and 74°5'19.2"W, 1368 m a.s.l. The type material was collected by Anisbeth Daza, Rosana Londoño and Sigmer Quiroga on 31 July 2015. The remainder of the material was collected by Anisbeth Daza, Rosana Londoño, Paula Sepúlveda and Sigmer Quiroga, on 21 March 2015.

########### Type repository.

The holotype and paratypes, and the additional specimen, are deposited in the Centro de Colecciones Biológicas de la Universidad del Magdalena (CBUMAG:TAR), Santa Marta, Colombia.

########### Species description.

Body uncoloured. A faint, though difficult to see, cuticular roughness visible on the caudal portion of the body. Eyes probably absent (definitely absent after mounting and no information record when mounted). Bucco-pharyngeal apparatus of the *Meplitumen*-type of Itaquasconinae model (as described above). Mouth terminal. A row of small teeth is present in the caudal portion of the buccal cavity (Fig. 3B, arrow). Pharyngeal bulb long, without apophyses but with two long, narrow rod-shaped macroplacoids (the second more than double the length of the first); microplacoid and septulum absent. Stylet furcae well developed but the caudal processes have non-swollen apices (Fig. 3D, arrow). Claws of the *Hypsibius*-type, with main branches provided with accessory points; these points are well developed on the internal claws, short and thin externally. At the base of the claws, a structure interpretable as a very thin lunule is present; other cuticular thickenings on the legs absent.

########### Choice of the holotype.

We found eight specimens and two exuviae with eggs and it is interesting to note that three specimens were small and of similar body size, whereas the others are markedly longer and certainly adults as demonstrated by the size of the exuviae with eggs. Unfortunately, we have not been able to establish the sex of the specimens; in particular, whether the smallest were three young, or new-born specimens, or were males. It is interesting to note that some metric characters of the smaller specimens are very similar to those of the larger specimens, while others were markedly different (see Table 1). If they are young or new-born examples, we would expect structures to have allometric growth, but these have more marked differences than that we have observed in other species. This makes us suspect the smaller specimens may be males. In any case, we have chosen the holotype among the larger, definitely adult, specimens.

########### Description of the holotype.

Body length 590 µm, uncoloured, cuticle with a faint, very difficult to see roughness in the caudal body portion (Fig. 5A, B, C arrows); as in other species, this cuticular roughness is not visible in some specimens. Eyes probably absent (definitely absent after mounting and no information record when mounted). Bucco-pharyngeal apparatus of the *Meplitumen*-type of Itaquasconinae model (Pilato and Binda 2010) (Figs 2 and 3), i.e., with the bucco-pharyngeal tube subdivided at the junction of the stylet supports into an anterior, almost rigid, buccal tube with spiral thickening (except the very anterior portion) (Fig. 2A, B, arrows ‘a’), and a posterior flexible portion with a more obvious spiral thickening. The mouth is terminal without peribuccal lamellae; peribuccal papulae probably present but requires confirmation. A row of small teeth present in the caudal portion of the buccal cavity (Fig. 3B, arrow). The AISM are flat, symmetrical with respect to the frontal plane, and with the caudal processes short and pointing sideways (Fig. 2A, arrow ‘c’). Bucco-pharyngeal tube 61.3 µm long, buccal tube 31.8 µm long (*pbf* index = *51.9*) and 8.9 µm wide externally (*pt* index = *28.0*), pharyngeal tube 29.5 µm long. No drop shaped thickening is present between buccal and pharyngeal tube (Figs 2A, B; 3A, B, D). The stylet furcae are well developed but have the caudal processes with non-swollen apices (Fig. 3D, arrow). Pharyngeal bulb (Fig. 3A, C) long, about 2.5 times its width (63.4 µm × 27.0 µm), without apophyses, but with two long, narrow, rod-shaped macroplacoids (Fig. 3A, B, C); first macroplacoid 10.9 µm long (*pt* = *34.3*), the second 29.0 µm long (*pt* = *91.2*); the entire placoid row is 40.7 µm long (*pt* = *128.0*); microplacoid and septulum absent.

Claws of the *Hypsibius*-type well developed (Fig. 6A, B, C) with widened extreme basal portion. Reliable claw measurements, as in many species, are challenging due to the claw orientation affecting the apparent length. The main branch of both external and internal claws with accessory points; more evident in the hind legs. In all legs those points are more developed on the internal claws (Fig. 6A, B, C) and very short and thin on the external claws; particularly those of the first three pair of legs where they are often almost invisible. At the base of the claws a structure interpretable as a very thin lunule is present (Fig. 6A, B, C arrow). Other cuticular thickenings on the legs absent.

########### Eggs.

We found two exuviae, one with 5 and the other with 6 smooth eggs.

########### Etymology.

The specific epithet refers to the term “Aluna” which in Ika, the native language of the Kogui (an Amerindian ethnic group inhabiting the Sierra Nevada de Santa Marta), means the non-visible or spiritual world. Aluna is a name in apposition.

########### Remarks.

As mentioned above, the three smaller specimens (Fig. 7A, B) show some metric differences from the larger. The most obvious differences are the *pt* values relative to the buccal tube width, the placoid length, and claws II, III (less remarkably claw IV) length. In all legs of all specimens the percent ratio between the main branch length and the total claw length are compatible (Table 1). This later character and the high number of important non-variable characters indicate they belong to *Meplitumenaluna* sp. nov., but the differences made us consider they may be very young individuals or males.

########### Differential diagnosis.

The particular combination of the characters of the bucco-pharyngeal apparatus distinguishes the new species from all known species of Itaquasconinae. It is possible that the presence of spiral thickening in the “buccal tube”, and the lateral orientation of the caudal processes of the AISM was missed in some previously described species. In this case, any species found with these characters should be transferred into the new genus *Meplitumen*, though this may result in *Meplitumenaluna* sp. nov. becoming a junior synonym. Therefore, to avoid this remote risk, we found it opportune to compare the new species with all the similar, known species of Itaquasconinae independently from the extension of the spiral thickening.

It is unnecessary to compare all *Itaquascon* species, as the presence in *Meplitumenaluna* sp. nov. of true macroplacoids, a more developed and differently shaped stylet furca (with longer branches) (Figs 3D arrow, and 4C arrow ‘a’), and more robust stylet supports (Figs 2A arrow ‘b’, and 4C arrow ‘b’), clearly separate these species. Also, comparison with *Astatumen* species is unnecessary as the presence in *Meplitumenaluna* sp. nov. of stylet supports, true macroplacoids (lacking in *Astatumen*), and a more developed and differently shaped stylet furcae (with longer branches) (Figs 3D arrow, and 4D arrow ‘a’), separates these taxa.

The differences with *Mesocrista* species are also clear, as Gąsiorek et al. (2016) confirmed the lack of spiral thickening in the buccal tube. In addition, in *Meplitumenaluna* sp. nov. the stylet supports are inserted exactly between the buccal and the pharyngel tube (Fig. 2A), whereas in the two known species of *Mesocrista* a short portion of tube without spiral thickening is present after the stylet supports insertion point (see Gąsiorek et al. 2016: fig. 2a, b, figs 5 a, b; 7c). Furthermore, in the new species the caudal processes of the stylet furcae do not have swollen apices (compare Fig. 3D arrow, and Fig. 4A arrow ‘a’), the microplacoid is absent, and cuticular bars on the legs are absent.

We carefully examined *Platicristaangustata* (Murray, 1905) and ascertained the absence of spiral thickening in the buccal tube wall (Fig. 4B). In addition, *Meplitumenaluna* sp. nov. differs from *Platicristaangustata* in having thinner stylet supports (Figs 2A arrow ‘b’, and 4B, arrow ‘b’), and thinner and longer claw secondary branches (compare Fig. 6A–C with 6D). The new species differs from *Platicristacheleusis* Kathman, 1990 by the lack of a polygonal pattern on the cuticle, and cuticular bars on the legs. It differs from *Platicristahorribilis* Kaczmarek & Michalczyk, 2003 in the claw shape, and by having the lunules (if they are such) on legs IV less developed and without teeth. *Meplitumenaluna* sp. nov. differs from *Platicristaitaquasconoide* (Durante Pasa & Maucci, 1975) by lacking the microplacoid, and a small basal spur on the claws of the hind legs. It is distinguished from *Platicristaramsayi* Marley, 2006 by the lack of cuticular bars on the legs.

*Platicristaaffine* (Mihelčič, 1951) is considered a *nomen dubium* (Ramazzotti and Maucci 1983, Dastych 1993, Marley 2006) and as the material studied by Mihelčič no longer exists, a comparison is impossible.

######### Phylogenetic analysis, discussion and conclusions

Preliminary discussion

The new genus *Meplitumen* is, in our opinion, particularly interesting as it possesses characters which allow us to imagine the evolutionary pathways that connect the five genera of Itaquasconinae*Mesocrista*, *Platicrista*, *Meplitumen* gen. nov., *Itaquascon* and *Astatumen* which constitute a homogeneous group inside that subfamily.

Bertolani et al. (2014) attributed to the subfamily Itaquasconinae the above four genera (five including our new genus), plus the genera *Adropion* Pilato, 1987; *Parascon* Pilato & Binda, 1987 and *Bindius* Pilato, 2009. Guil et al. (2014) described the genus *Sarascon*, and attributed it to the same subfamily. These additional four genera possess some characters that are similar to the five genera we have studied but differ in key characters. The AISM for *Adropion* and *Bindius* are very different being hock-shaped, very thin in dorso-ventral view and not flat. *Parascon* clearly differs in having a short pharyngeal tube without spiral thickening, and *Sarascon* has the same characters as *Parascon*.

Above all, the absence of molecular data for *Bindius* and *Parascon* and the fact that we have not had the good fortune to examine *Sarascon*, we prefer in this paper to concentrate our attention on the five genera *Mesocrista*, *Platicrista*, *Meplitumen*, *Itaquascon* and *Astatumen* that certainly, within the family Itaquasconinae, constitute a homogeneous group.

Gąsiorek et al. (2016), in the revision of *Mesocrista*, refrained from putting forward a hypothesis about the phylogenetic relationships between the known genera *Mesocrista*, *Platicrista*, *Itaquascon*, and *Astatumen*. In their defence, they wrote (page 22), “As shown and noted above, the scarcity of molecular data for Itaquasconinae prevents a through phylogenetic analysis of the subfamily”. They accepted that, “given that a close morphological similarity of many Itaquasconinae genera might suggest close ancestry, 18S rRNA, being a relatively conservative marker, may not be most suitable for resolving relationships within the subfamily”. In conclusion, Gąsiorek et al. (2016) suggested that there was a need to, “test whether analyses of fragments exhibiting higher mutation rates, such as 28S rRNA and COI would produce the same topologies or would corroborate with classic taxonomy”. We do not deny the importance of molecular data but we also think that, as a result of the description of *Meplitumen*, morphology already permit us to propose a hypothesis about the phylogenetic relationships between the five above mentioned genera of Itaquasconinae.

Taking into account the morphology of the Hypsibiidae, some easily recognisable evolutionary tendencies can be noticed in the group of genera we are considering.

The first regards the shape of the AISM, which tend to become wide and flat and whose caudal processes only in *Mesocrista* point postero-laterally, as in all the other Parachela, while in *Platicrista*, *Meplitumen*, *Itaquascon* and *Astatumen* they point laterally. These processes are definitely more robust in *Mesocrista* and *Meplitumen*, while they are thinner in *Platicrista*, and extremely thin, even flexible, in *Itaquascon* and in *Astatumen*.

A second evolutionary trend regards the shape and size of the stylet furcae. In *Mesocrista* only are the furca processes well developed and with swollen apices (Fig. 4A, arrow ‘a’). However, the furcae general shape is not exactly the same as *Diphascon*, *Adropion*, and the other non-ItaquasconinaeHypsibiidae; the furcae body being slightly reduced in *Mesocrista*. In addition to this, in *Platicrista* (Fig. 4B arrow ‘a’), and *Meplitumen* (Fig. 3D, arrow) the apices are not swollen, and in *Itaquascon* and *Astatumen* the whole processes tend to be very short or even almost missing, and as a result the entire furcae are very small (Figs 4: C arrow ‘a’; and D, arrow ‘a’).

A third evolutionary trend regards the stylet supports, which are normally developed in *Mesocrista*, *Meplitumen* and *Platicrista*, very thin in *Itaquascon* (Fig. 4C, arrow ‘b’), and absent in *Astatumen* (Fig. 4D).

The fourth evolutionary direction is that of the placoids, which are present, long and slender in *Mesocrista*, *Meplitumen*, and *Platicrista*. In *Itaquascon* and *Astatumen* these are substituted by a long, simple cuticular thickening, which can also be (or seem to be) absent in some species.

Some of these evolutionary tendencies are also recognisable in the genera *Bindius*, *Parascon* and *Sarascon* but, as mentioned above, we are not considering these three genera in this paper.

######### Phylogenetic analysis and its discussion

**Figure 8.**
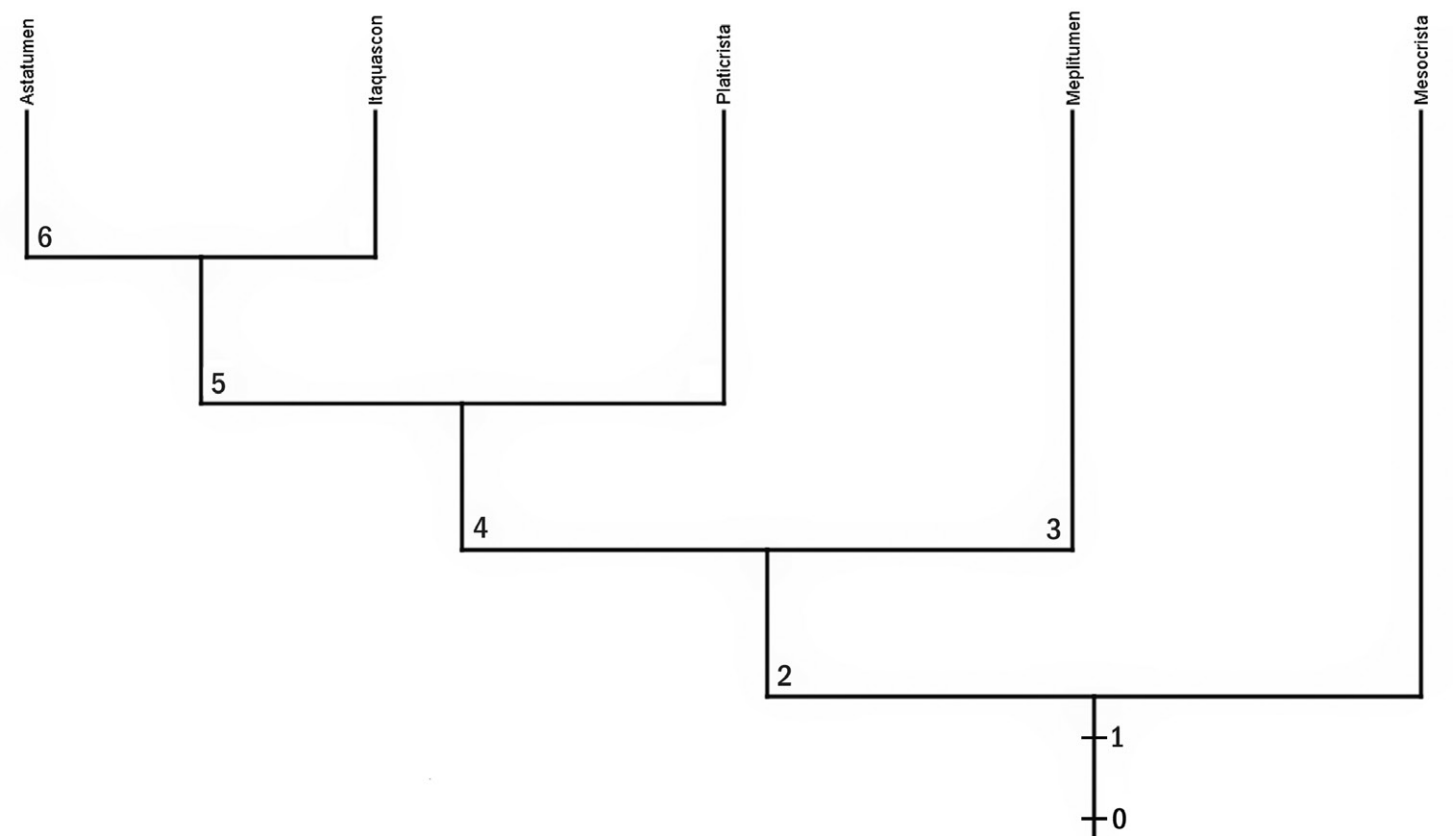
Most supported phylogenetic tree, according to character matrix in Table 2, of the genera *Mesocrista*, *Platicrista*, *Itaquascon*, *Astatumen* and *Meplitumen* (phylogenetic tree values: consistence index = 0.91; retention index = 0.83). **0** = Ancestor with ordinary AISM (i.e. not wide and flat and with caudal processes pointing postero-laterally), stylet supports present, spiral thickening present only after the stylet supports, ordinary stylet furcae and placoids. **1** = AISM became wide and flat, stylet furcae reduced slightly, placoids became elongated; *Mesocrista* maintained these characters. **2** = Caudal processes of the AISM pointed laterally; apices of the caudal processes of the stylet furcae became non-swollen. **3** = appearance of spiral thickening also on the anterior buccal tube; this line gave rise to *Meplitumen*. **4** = AISM became wider and their caudal processes reduced; *Platicrista* maintained these characters. **5** = Stylet furcae small and with reduced caudal processes, placoids reduced to a single, very thin, bar or absent, stylet support slendered; *Itaquascon* maintained these characters. **6** = Stylet supports disappearance, spiral thickening present also on the anterior buccal tube; this line gave rise to *Astatumen*.

A parsimony analysis of the character matrix shown in Table 2 resulted in a best supported tree with consistence index = 0.91 and retention index = 0.83 (Fig. 8).

**Table 2.** Character matrix used for the phylogenetic analysis. AISM = apophyses for the insertion of the stylet muscles.

	**AISM shape**	**AISM caudal process development**	**AISM caudal process orientation**	**Stylet furcae**	**Stylet furcae apices**	**Anterior tube spiral thickening**	**Placoids**	**Stylet supports**
* Mesocrista *	moderately wide	robust	postero-lateral	“big” with developed branches	swollen	absent	present (slender)	present (normal)
* Meplitumen *	moderately wide	robust	lateral	“big” with developed branches	non-swollen	present	present (slender)	present (normal)
* Platicrista *	very wide	thin	lateral	“big” with developed branches	non-swollen	absent	present (slender)	present (normal)
* Itaquascon *	very wide	extremely thin, even flexible	lateral	small with reduced branches	non-swollen	absent	reduced to a thin bar or absent	present (slendered)
* Astatumen *	very wide	extremely thin, even flexible	lateral	small with reduced branches	non-swollen	present	reduced to a thin bar or absent	absent

*Mesocrista* is the genus which most closely resembles the other Hypsibiidae (i.e. ancestor “0” in Fig. 8). This places it as the most basal of the group we are considering, but already some of the four discussed evolutionary tendencies have started to be expressed (i.e., AISM becoming wide and flat, slight stylet furcae reduction, placoid elongation). The gradual expression of all four evolutionary tendencies in the other four genera make them more derived. It is worth mentioning that our hypothesis about ancestor “0” of Fig. 8, is actually based on the characters present in all the other Hypsibiidae (AISM not wide and flat and with caudal processes pointing postero-laterally, ordinary stylet supports, stylet furcae and placoids), or at least in the *Diphascon*, *Adropion*, Pilatobiinae Bertolani, Guidetti, Marchioro, Altiero, Rebecchi & Cesari, 2014, and Diphasconinae Dastych, 1992 (spiral thickening present only after the stylet supports).

In choosing the proposed phylogenetic tree, we have been careful not to allow character reversals. However, the spiral thickening on the anterior buccal tube is present in two separate branches of the tree (in *Meplitumen* and *Astatumen*), which we hypothesise is not a reversal but the character appearing twice, independently, in those two branches. This is the only weak point of the proposed tree but we want to stress that any change in the tree aimed at correcting this situation invariably produces multiple reversals in other characters. This double appearance of the anterior buccal spiral thickening character in *Meplitumen* and *Astatumen* might be explained by hypothesising that their common ancestor already possessed a genetic prerequisite (to form spiral thickening) thus requiring only one, or few mutations, to express the character.

In *Mesocrista* the annulation of the pharyngeal tube was described as double (Gąsiorek et al. 2016: fig. 2d). Future investigations may reveal this character is also present in *Platicrista*, *Meplitumen*, *Itaquascon*, and *Astatumen*, in which case the phylogenetic relationship would be reinforced. However, if simple annulation (non-double) were confirmed in these genera, this would not necessarily disprove the affinity. It is possible all these genera were derived from an ancestor similar to *Mesocrista* but with simple annulation, and that *Mesocrista* acquired, individually, the double annulation.

## Conclusions

It is clear that in all cases, any attempt to reconstruct phylogenetic relationships requires making a hypothesis that can be tested, and which might give rise to doubts or different opinions. We think that a hypothesis can be proposed when clear characters and evolutionary tendencies are observed in known members of a taxon (for which the phylogeny is totally unknown). Such a proposed hypothesis is justified and needs to be brought to the attention of the scientific community, while awaiting new data to confirm or create a new, more convincing, phylogenetic reconstruction.

The present work also adds value to the biodiversity of Colombia, with a new species and a new genus that, at least for the moment, result endemic for the country. It is worth mentioning that we are about to publish another new genus (of a different family) from Colombia, proving the high potential of these investigations, since to date very little is known about the tardigradological fauna of the country. This encourages us to go on with our studies.

## Supplementary Material

XML Treatment for Meplitumen

XML Treatment for Meplitumen aluna

## Footnotes

in Marley et al. 2011; amended by Bertolani et al. 2014 and Gąsiorek et al. 2018

amended by Bertolani et al. 2014 and Gąsiorek et al. 2018

sensu Pilato 1987, amended by Bertolani et al. 2014

## Appendix 1

The presence in *Meplitumen* of spiral thickening to almost the whole length of the bucco-pharyngeal tube gives rise to a problem of interpretation, and, as a consequence, terminology of the subdivisions of the *Astatumen* bucco-pharyngeal tube. In all tardigrades with bucco-pharyngeal tube divided into a rigid anterior portion without spiral thickening and a flexible posterior portion with spiral thickening, the spiral thickening and the stylet supports insertion point have been used to differentiate between the anterior buccal tube and the posterior “pharyngeal tube”. As a consequence, in *Astatumen*, which lacks stylet supports, only the spiral thickening has been taken as the reference point, and therefore the buccal tube has been considered to be only the very anterior portion where the AISM lie. However, the distinctive situation we have encountered in *Meplitumen* made us realise that the short portion defined buccal tube in *Astatumen* does not correspond to the equivalent buccal tube of the other genera, but only to its anterior part. The absence of stylet supports in *Astatumen* prevents the location of the exact border between what in other genera is defined buccal and pharyngeal tube. Having clarified this situation it would be necessary to modify the terminology, as well as the evaluation of the length of the buccal tube, to redefine the short anterior portion of buccal tube without spiral thickening. Nevertheless, the problem of fixing the exact border between the real buccal tube and pharyngeal tube, and calculating the exact lengths, would remain unsolved. In conclusion, we find it is important to have clarified the morphological and phylogenetic meaning of these structures, but for practical reasons we think that it would not be opportune to change the terminology.
